# A cost-benefit/cost-effectiveness analysis of proposed supervised injection facilities in Ottawa, Canada

**DOI:** 10.1186/1747-597X-9-31

**Published:** 2014-08-04

**Authors:** Ehsan Jozaghi, Andrew A Reid, Martin A Andresen, Alexandre Juneau

**Affiliations:** 1 School of Criminology, Simon Fraser University, 8888 University Drive, Burnaby, British Columbia V5A 1S6, Canada

**Keywords:** Supervised injection facilities, HIV, HCV

## Abstract

**Background:**

Supervised injection facilities (SIFs) are venues where people who inject drugs (PWID) have access to a clean and medically supervised environment in which they can safely inject their own illicit drugs. There is currently only one legal SIF in North America: Insite in Vancouver, British Columbia, Canada. The responses and feedback generated by the evaluations of Insite in Vancouver have been overwhelmingly positive. This study assesses whether the above mentioned facility in the Downtown Eastside of Vancouver needs to be expanded to other locations, more specifically that of Canada’s capital city, Ottawa.

**Methods:**

The current study is aimed at contributing to the existing literature on health policy by conducting cost-benefit and cost-effective analyses for the opening of SIFs in Ottawa, Ontario. In particular, the costs of operating numerous SIFs in Ottawa was compared to the savings incurred; this was done after accounting for the prevention of new HIV and Hepatitis C (HCV) infections. To ensure accuracy, two distinct mathematical models and a sensitivity analysis were employed.

**Results:**

The sensitivity analyses conducted with the models reveals the potential for SIFs in Ottawa to be a fiscally responsible harm reduction strategy for the prevention of HCV cases – when considered independently. With a baseline sharing rate of 19%, the cumulative annual cost model supported the establishment of two SIFs and the marginal annual cost model supported the establishment of a single SIF. More often, the prevention of HIV or HCV alone were not sufficient to justify the establishment cost-effectiveness; rather, only when both HIV and HCV are considered does sufficient economic support became apparent.

**Conclusions:**

Funded supervised injection facilities in Ottawa appear to be an efficient and effective use of financial resources in the public health domain.

## Background

The spread of infectious diseases among people who inject drugs (PWID) is a major public health issue. Research studies conducted throughout the developed and developing world have found that diseases such as human immunodeficiency virus/acquired immunodeficiency syndrome (HIV/AIDS) and hepatitis C virus (HCV) are some of the leading causes of death among PWID who share needles and engage in other unsafe practices [1]. According to the World Health Organisation, there are approximately 16 million people who inject drugs worldwide, and 3 million of those PWID are suffering from HIV [2]. The most recent data in Canada indicates that in the year 2002, there were almost 1700 deaths related to illegal drug use. Moreover, that year, there were 87 AIDS deaths caused by illegal drug use and it was found that 70 percent of the new HCV infections could be traced to illegal drug use [3]. In an effort to control this public health issue, needle exchange programmes (NEP) have become one of the most established means of harm reduction among PWID and have proven to have a positive impact on public health [2-7].

One harm reduction strategy that has emerged – to address this public health issue – is the supervised injection facility (SIF). SIFs are venues where people who inject drugs have access to a clean and medically supervised environment in which they can safely inject their own illicit drugs. There is currently only one legal SIF in North America – Insite in Vancouver, British Columbia, Canada. Since beginning its operations in 2003, this facility has been examined in over 50 peer-reviewed studies. The vast majority of these studies have had positive conclusions. For example, there has been a reduction in overdose fatalities, needle sharing, and an improvement in public order [8-13]. Many of these studies’ analyses have shown Insite to be cost-effective: saving taxpayers considerable money through the prevention of new HIV and HCV infections as well as reducing risky injection behaviours [8,14-17]. Given these facts, it has been proposed that the use of SIFs should be expanded to other large cities in Canada, such as Victoria, Montreal and Ottawa [14,18,19].

A report published in 2004 citing personal communication with Professor Robert Remis, stated that the PWID population in Ottawa, Ontario comprised between 3,000 and 5,000 individuals [20]. Ottawa’s PWID population currently has some of the highest rates of new HIV and HCV infections [21]. Studies have estimated HIV prevalence ranging from 11% to 21% and HCV prevalence between 55% and 76% [21,22]. These rates are both higher than those found in Toronto, which is Canada’s largest city with a population of over 6 million persons residing in the Greater Toronto Area [21]. Leonard et al. found that among Ottawa PWID, 37% of women and 31% of men said they injected with used needles in the six months preceding their interview with the researchers [22]. More troubling is the fact that the rates of infection and unsafe injection practices are so high despite the widespread use of NEPs and other harm reduction strategies.

Given this data, it can be argued that new strategies should be considered to help reduce these rates and prevent new infections from occurring in PWID. In effect, The Sandy Hill Community Health Centre and partners planned to submit an exemption application for many years but it has been delayed because both Mayor Jim Watson and police Chief Charles Bordeleau have opposed the idea. Dr. Mark Tyndall, chief of infectious diseases at the Ottawa Hospital, told an audience at a recent rally at the Canadian Parliament hill that “a site would send a message of care to addicts and reduce harm. Tyndall said many drug users in Ottawa aren’t accessing existing services, and says a site would connect them” [23], p. 1. Moreover, a group of community members that advocate for the opening of safer consumption sites in Ottawa for PWID has been formed and they opine that “the most effective response to problematic drug use includes harm reduction, expanded social and health care services, preventative measures to address communicable diseases, and evidence-based drug policies” [24], p.1.

Furthermore, a team of University of Toronto researchers concluded in a recent report that three SIFs in Toronto and two SIFs in Ottawa would prevent the spread of HCV and HIV, save money, and reduce sharing of needles within the PWID population [21]. However, the difference between this costing study and Bayoumi and Strike’s [21] study is the mathematical model and sensitivity analysis used. Bayoumi and Strike’s [21] study, similar to Bayoumi and Zaric’s [15], used a complex dynamic compartmental simulation model, that incorporated factors like co-infections, smoking related drug use, and the proportion receiving methadone. In terms of HCV, their analysis of Toronto accounted for 15 to 20 preventive cases, thereby resulting in savings of a total of CAN $47,489 for the first facility [21]. In Ottawa, the savings are more modest for HCV, predicting a cost-savings of $18,591 [21].

Along the lines of the Bayoumi and Strike [21] report, this paper examines if opening SIFs in Ottawa would be an effective use of fiscal resources – based on the combined cost-savings of co-morbidity infections such as HIV and HCV. Though recent studies have shown that the establishment of SIFs is cost-effective, particularly in a Canadian context [8,14-18,21], it is important to consider the different base rates of HIV and HCV infection, as well as other model parameters such as needle sharing rates, that are likely to impact whether SIFs are cost-effective or not. This is of importance because the specific PWID characteristics are different in different areas. As a result of this, different base rates of both HIV and HCV infections are likely to determine whether SIFs are cost effective, as shown in the studies listed above. Specifically, this is done by conducting cost-benefit and cost-effectiveness analyses for operating a SIF in the Ottawa region. The costs of operating a SIF in Ottawa will be compared to the savings incurred by the healthcare system after accounting for the prevention of new HIV and HCV infections.

### Related research

Closely related to this study is a burgeoning body of recent research that has evaluated the economic viability of Vancouver’s Insite facility. Much of this research has investigated the impact of the SIF in reducing the number of HIV and HCV infections. However, it should be noted that such a relationship has not been demonstrated in any definitive manner in the scientific research conducted. As discussed below, mathematical modelling approaches are used to estimate expected outcomes, and not to count actual changes. This lack of definitive scientific evidence is in part due to the same size constraints, the very high incidence rate of HCV among PWID, and the difficulty in obtaining ethical approval for such studies. Though this is not scientific evidence, these models serve as excellent tools to identify what kind of changes we can expect when public health policy is implemented.

The first of these studies was published in 2008. In that study, Bayoumi and Zaric [15] projected new HIV and HCV infections for the City of Vancouver over a 10-year period. Using a complex dynamic compartmental simulation model, the study made projections with and without the Insite facility. Results estimated that over the 10-year time period, 1191 new HIV and 54 new HCV cases would be averted with the implementation and use of the SIF [15]. As a result, considering the average annual number of new HIV cases averted (120), the lifetime cost of a new HIV infection (CDN$210 555) [16,25], and the cost of operations for the SIF portion of Insite (CDN $1.5 million) [18], the SIF would yield annual savings of CDN $25 million – at a benefit-cost ratio of 16.84.

It has been argued that a more realistic economic assessment was completed by Des Jarlais et al. [26] where Insite was estimated to prevent 20 – 30 new cases of HIV each year [14]. Using that figure along with the same lifetime cost of a new HIV infection (CDN $210 555) and operational cost of Insite (CDN $1.5 million), it was estimated that benefit-cost ratios varied from 2.81 and 4.21 [26]. These, more conservative estimates, are not nearly as compelling as those found by Bayoumi and Zaric [15] but nevertheless sustain sufficient support for the continued operation of Insite in the City of Vancouver.

Adopting a different methodological approach, Andresen and Boyd [8] conducted cost-benefit and cost-effectiveness analyses of Insite that used four separate mathematical models to assess the economic impact of preventing new HIV infections each year. Results of their study revealed that between 19 and 57 new cases may be averted depending on the model selected, in addition to an average of 35 new cases being prevented every year [8]. With respect to the benefit-cost analysis, results were comparable to those of Des Jarlais et al. [26], who considered credible changes in HIV infection rates, with ratios ranging from 1.94 to 5.8 and an average of 3.56. While these results too, support the economic rationale for the operation of Insite, more recent studies have revealed conflicting results.

Pinkerton [16,17], for example, used Kaplan’s [5,27] needle circulation theory to demonstrate that while Insite – as a whole – may be very cost-effective, much of its effectiveness is attributable to its needle exchange program. In fact, Pinkerton [16,17] concluded that the SIF component alone does very little to prevent new cases of HIV. Specifically, of the 83.5 new HIV cases averted each year in the 2010 study, only 2.8 may be attributed to the SIF [16]. Similarly, the 2011 study revealed that a mere 4 to 8 new HIV cases are averted each year [17]. With greatly reduced benefit-cost ratios of 0.37 [16] and 0.8 [17], respectively, these results suggest that the SIF portion of Insite is not a practical harm reduction option, at least in economic terms.

While one may question the discrepancy in results between this collection of studies, Andresen and Jozaghi [14] note that the differences are attributable to the choices of variables in the models presented. As such, “Pinkerton (2010, 2011) does not consider behavioural changes of PWID with regard to needle-sharing in his models” [14], p4 while the Bayoumi and Zaric [15] and Andresen and Boyd [8] studies do. Because previous research has shown that Insite users have a lower rate of needle-sharing than non-Insite users [12], the Bayoumi and Zaric [15] and the Andresen and Boyd [8] approaches should be considered as more accurate representations of actual change in PWID behavior.

With sufficient (economic) evidence supporting the continued operation of Insite, Andresen and Jozaghi [14] posed another crucial question: should Insite be expanded in the Downtown Eastside community of Vancouver? Using a mathematical model to predict the number of new HIV infections, Andresen and Jozaghi [14] assessed the viability of expanding the operation of the Insite facility within Vancouver – both in terms of its individual operating capacity and the potential for additional SIFs. They found that although increased hours and extended service delivery by Insite itself would result in modest benefits, the addition of further SIFs in other geographic areas of the city would have a far greater and a more justifiable economical impact [14]. Specifically, the benefit-cost ratios supported the expansion of as many as five additional SIFs.

Related to research on the expansion of SIFs, Jozaghi et al. [18] conducted a study to assess the economic viability of opening SIFs in the city of Montreal, Quebec, Canada. Adopting a more comprehensive research design than the one employed in Andresen and Jozaghi [14], the authors estimated the number of new HIV and HCV infections that would be prevented with the introduction of SIFs in Montreal. Accounting for the prevention of each of these types of harmful diseases, they found that an annual net cost savings of CDN$686 000 (HIV) and CDN$800 000 (HCV) would be expected for each additional SIF [18]. Including a variety of SIF operation scenarios to assess the threshold for diminishing returns, they noted that the cost saving figures could be expected with expansions that extend to a maximum of three SIFs. See table 1 for a summary of costing studies conducted on SIFs.

**Table 1:** Summary of costing studies conducted on SIFs

**Study**	**Cost-effectiveness model**	**Variables included**	**Findings**
The cost-effectiveness of Vancouver’s supervised injection facility (Bayoumi AM, Zaric GS). 2008 [15]	Dynamic compartmental model; 10-year time horizon	▪ IDUs, non-users, persons with HIV and HCV, those with combinations of these states	▪ Over 10-year time horizon, the introduction of a SIF in Vancouver would prevent 1191 cases of HIV and 54 cases of HCV
		▪ Sexual transmission, transmission through needle sharing	▪ Negative net cost of SIF
		▪ Population, population shifts	▪ Vancouver SIF would save money and increase life expectancy
		▪ Annual costs	
A cost–benefit and cost-effectiveness analysis of Vancouver’s supervised injection facility (Andresen MA, Boyd NT). 2010 [8]	Mathematical modelling	▪ Number of IDUs in population, number of sharing partners, participation rate at Insite	▪ Insite has a positive impact on the health outcomes of IDU population
		▪ Number of needles used per client-year, number of needles in circulation, percentage of HIV infected needles, percentage of needles not cleaned	▪ Vancouver SIF prevents 35 new cases of HIV and almost 3 deaths annually.
		▪ Number and rate of shared injections per year	▪ Provides societal benefit in excess of $6 million per year after programme costs are taken into account
		▪ Probability of HIV infection from a single injection, cumulative probability of HIV infection, HIV prevalence rate	▪ Average benefit-cost ratio of 5.12:1
		▪ Reduction of risk from participation	
Is Vancouver Canada’s supervised injection facility cost-saving? (Pinkerton SD). 2010 [16]	Mathematical modelling 1-year time frame	▪ IDUs living in Vancouver	▪ If Insite were closed, HIV infections among Vancouver IDU would increase from 179.3 (1.6% annual incidence) to 262.8 (2.3% incidence)
		▪ Prevalence of HIV infection (%), annual incidence of HIV infection (%)	▪ This represents a difference of 83.5 infections per year
		▪ Injections per IDU, per year, injections with borrowed syringes (%), supervised facility injections, per year	▪ These preventable infections would be associated with $17.6 million in life-time HIV-related medical costs
		▪ Syringes distributed in Vancouver, per year, syringes distributed by Insite SEP, syringes distributed by	▪ The savings in cost exceeds Insite’s annual operating costs of approximately $3 million.
		▪ non-Insite sources	▪ Most infections were prevented thanks to Insite’s syringe exchange program, which would prevent 80.7 infections
		▪ Annual operating cost (Canadian $)	
How many HIV infections are prevented by Vancouver Canada’s supervised injection facility? (Pinkerton SD). 2011 [17]	Mathematical modelling	▪ Number of IDUs	▪ Vancouver SIF prevents approximately 5–6 infections per year, with a range of 4–8 prevented infections
		▪ HIV prevalence, per injection transmission rate	▪ Insite SIF reduces HIV incidence among DTES IDU by 6-11%
		▪ Incidence rate without Insite, incidence reduction	
		▪ Syringes contaminated with HIV, decontamination rate	
		▪ Borrows per IDU per year with Insite, reduction in number of borrows	
		▪ SIF injections per IDU per year	
Potential role of safer injection facilities in reducing HIV and Hepatitis C infections and overdose mortality in the United States (Semaan S, Fleming P, Worrell C, Stolp H, Baack B, Miller M). 2011 [28]	Six-factor Kass ethical framework for public health programs (goals, effectiveness, concerns, minimization of concerns, fair implementation, and balancing of benefits and concerns)	▪ Public health goals of SIFs and need for SIFs	▪ SIFs provide settings and public health interventions that support safer behaviors and aim to prevent and reduce HIV, HBV and HCV infections, infection disparities, overdose mortality, and injection-related bacterial infections
		▪ Effectiveness of SIFs in achieving public health goals	▪ SIFs are cost-saving and cost-effective, prevent accidental needle-stick injuries in community members, and reduce public nuisance and litter
		▪ Potential concerns	▪ SIFs provide unique and complimentary services to other public health interventions that promise to improve the health of PWIDs and the public order and safety of communities blighted by public injection
		▪ Minimization of concerns and role of other programs	▪ SIFs provide sterile injection and drug preparation equipment at time of injection, a safe and medically attended environment, and on-site counseling or referrals to health and social services, including addiction treatment and housing
		▪ Fair implementation of important ethical and contextual factors that influence the ethical deliberations and operational aspects of public health programs	
The point of diminishing returns: an examination of expanding Vancouver’s Insite (Andresen MA, Jozaghi E). 2012 [14]	Mathematical modelling (Jacobs et al. (1999) mathematical model)	▪ Expanding Insite’s hours of operation	▪ Insite operational for 18 hours predicts that 22 new cases of HIV are averted annually
		▪ Increasing the number of SIFs	▪ Insite is cost-saving. The cost-benefit ratio is 3.09. The number of new HIV infections averted, and the associated cost-savings, are more than enough to cover Insite’s annual operating costs
		▪ Proportion of IDUs HIV-negative	▪ Insite operational for 24 hours does not prevent any new HIV infections
		▪ Number of needles in circulation	▪ Expansions of Insite only prevent 1 or 2 additional new cases of HIV infection
		▪ Rate of needle-sharing	
		▪ Percentage of needles not cleaned	
		▪ Proportion of IDUs HIV-positive	
		▪ Probability of HIV infection from single injection	
		▪ Number of sharing partners	
A cost-benefit/cost-effectiveness analysis of proposed supervised injection facilities in Montreal, Canada (Jozaghi E, Reid AA, Andresen MA). 2013 [18]	Mathematical modelling using secondary data	▪ Proportion of IDUs HIV-negative, proportion of IDUs HIV-positive, proportion of IDUs HCV-negative, proportion of IDUs HCV-positive	▪ Increasing scope of SIFs through site expansion would result in 14–53 fewer HIV and 84–327 fewer HCV cases annually. The marginal range would result in 5–14 fewer HIV and 33–84 fewer HCV cases annually
		▪ Number of needles in circulation, percentage of needles not cleaned, rate of needle sharing	▪ Establishing SIFs in Montreal will benefit the health care system and expanding SIFs would be a fiscally responsible course of action
		▪ Probability of HIV infections from a single injection, probability of HCV infection from single injection	▪ With the HIV and HCV cases averted, SIFs in Montreal would be cost-saving
		▪ Number of sharing partners	

In addition to these economic arguments in support of SIFs, Semaan et al. [28] assess the broader role of SIFs in reducing HIV and HCV infections as well as overdose mortality. In this paper, the authors considered ethical, operational, and public health issues while arguing for the expansion of SIFs into the United States. With all of these positive assessments, a question arises: Are SIFs an economically viable option for other cities? With respect to the focus of this study specifically: are SIFs a cost-effective harm reduction strategy for Ottawa, Ontario where the rates of HIV and HCV have been estimated to be higher than those in other major cities in Canada [21]? Ottawa is of particular interest because, as noted above, this city is considering the establishment of a SIF and has issues related to PWIDs that are different from those in Vancouver.

### Data and methods

As shown in Table 2, the data for both the mathematical models was derived from secondary sources collected from both published and unpublished studies in 2013. Of the twelve variables used in the analysis below, six of them were obtained from research directly related to the PWID population in Ottawa; the remaining variable values were obtained from relevant research articles that have been well-received by researchers in this field.

**Table 2:** Sources for variables used in mathematical modeling

**Variable**	**Value**	**Source**
Proportion of PWID HIV- (**I**)	88.00%	Bayoumi & Strike [21]; Pilon et al. [29]
Proportion of PWID HCV- (**I**)	39.40%	Bayoumi & Strike [21]; Pilon et al. [29]
Rate of Needle sharing (**s**) or (**λ**)	14%	Bayoumi & Strike [21]
Number of needles in circulation (**N**)	837931	City of Ottawa [30]
Percentage of needles not cleaned (**d**)	17.00%	Kaplan and O’Keefe [5]; Jacobs et al. [31]
Probability of HIV infections from a single injection (**t**) or (**α**)	0.67%	Kaplan and O’Keefe [5]
Probability of HCV infections from a single injection (**t**)	3%	Gore & Bird [32]
Number of sharing partners (**m**)	1.38	Jacobs et al. [31]
Proportion of PWID HIV + (**q**) or (**π**)	12.00%	Bayoumi & Strike [21]; Pilon et al. [29]
Proportion of PWID HCV + (**q**)	60.60%	Bayoumi & Strike [21]; Pilon et al. [29]
Proportion of HIV or HCV infected needles (**β**)	40.50%	Kaplan and O’Keefe [5]
Probability of needles cleaned (**θ**)	83%	Kaplan and O’Keefe [5]; Jacobs et al. [21]

Modeling a SIF such as Insite must be done with caution in order to have credible results. Unlike previous work on Insite – that has considered expansions in terms of hours of operation and subsequent facilities – we only consider the establishment of a 24-hour SIF and its subsequent expansions on both HIV and HCV infections. We use a 24-hour operation because this has proved to be a reliable operation framework in some of our previous research work [14,18]. If the reader wishes to consider an 18-hour operation, the economic benefits should be multiplied by 0.75. These changes are investigated using the Jacobs et al. [31] and Kaplan and O’Keefe [5] models, described in detail below; the primary variable of interest that is influenced by the presence of a SIF is a modification of the rate of needle sharing. Because none of the injections within the SIF are shared injections, its presence decreases the rate of needle sharing in the PWID population, with the rate of needle sharing sequentially changing (decreasing) as more SIFs are added to the mathematical model. However, these models are static, even when considering changes in the rate of needle sharing: over time the presence of a SIF will impact the proportion of PWID that are HIV + and HCV+. Consequently, with our models only considering changes in the rate of needle sharing, our analyses under-estimates the impact of subsequent SIF expansions.

There are many choices of mathematical models to use in such an investigation. As discussed above, we employ the modified versions of the first model (Jacobs et al. [31]) and the second (Kaplan and O’Keefe [5]) mathematical model of needle exchange programs to address the changes in the rate of needle sharing, influenced by the establishment of a SIF. The second model (Kaplan and O’Keefe [5]), and variations thereof, has been used often in the NEP evaluation literature in addition to being used in the SIF evaluation literature [8]. The first model (Jacobs et al. [31]) has been particularly instructive for cost-benefit and cost-effectiveness analyses in the context of Vancouver’s SIF, Insite, and expansions to other areas within Canada [8,14,18]. Moreover, the first model has been shown to produce estimates of HIV infection in the PWID population that are very similar to known data in the Canadian context [14]. Within each of these mathematical models we also employ behavioural changes in PWID. These behavioural changes relate to PWID needle sharing behaviour outside of the SIF. Kerr et al. [13] and Bravo et al. [33] found that PWID who used the Vancouver SIF also reduced their needle-sharing activities significantly outside of Insite, with an odd ratio of 0.30. This has been incorporated into a number of cost-benefit and cost-effectiveness analyses for SIFs [8,14,15,18,28]. Because of its widespread use for Insite, and its empirical evidence [13,33] we incorporate such behavioural change for Ottawa in the analyses below.

The first model is estimated as follows:

$$\mathrm{New}\;\mathrm{HIV}/\mathrm{HCV}\;\mathrm{infections}\;=\;\mathrm{INsd}\left[1-{\left(1-\mathrm{qt}\right)}^{m}\right]$$

where *I* is the proportion of PWID that are HIV- (HCV-), *N* is the number of needles in circulation, *s* is the rate of needle sharing, *d* is the percent of needles not cleaned, *q* is the proportion of PWID that are HIV + (HCV+), *t* is the probability of an HIV (HCV) infection from a single injection, and *m* is the average number of sharing partners. It should be noted that needle cleaning is widely understood to be partially ineffective, but is retained to keep the model intact because of its accurate HIV infection estimates, as stated above. The values for these parameters (and their sources) are shown in Table 1. In order to estimate the impact of the SIF on new cases of HIV and HCV infections, the rate of needle sharing variable is manipulated: no shared injections are performed within the SIF, and because of the behavioural change regarding needle sharing, there are fewer shared injections outside of the SIF, except in the case of those who are users of the SIF.

The second model is estimated as follows:

$$\mathrm{New}\;\mathrm{HIV}\;\mathrm{infection}\;\mathrm{rate}=\left(1-\pi \right)\lambda \left(1-\theta \right)\beta \alpha$$

where π is HIV prevalence rate, λ is the rate of needle sharing, θ is the percentage of needles not cleaned, β is the percentage of HIV infected needles, and α is the probability of HIV infection from single injection. As with the Jacobs et al. [31] mathematical model, the values for these parameters (and their sources) are shown in Table 2. And the rate of needle sharing variable is manipulated in the same manner. Based on research conducted in Ottawa, our baseline percentage of needle sharing is 14 [21]. However, in the interests of undertaking a sensitivity analysis – in addition to employing two different mathematical models – we increase and decrease the values of this variable by 5 percent (19 and 9 percent, respectively) and recalculate the model results.

To ensure reliability, two distinct mathematical models and a sensitivity analysis were employed. Cost-effectiveness analysis is one of the main tools of economic evaluation and the heart of every economic analysis is a sensitivity analysis. There are a number of assumptions that are taken into account in every economic analysis, some of which may not be accurate, thereby introducing elements of uncertainty. This is especially true when predicting the cost of a hypothetical program. Therefore, sensitivity analysis and using additional mathematical models as a secondary form of sensitivity analysis “formalizes ways to measure and evaluate this uncertainty. Various researchers have made note of particular sources of uncertainties that may arise in costing studies [34], p. 297. In this work, we employ two models and a sensitivity analysis to account for all sources of uncertainty. Additionally, the behavioural change that impacts the rate of needle sharing is only applied to the establishment of the first two SIFs. This is done to generate more conservative results. If the behavioural change is applied in the same manner to each subsequent SIF established, it can be implicitly assumed that each SIF attracts a completely new set of clientele. This is an unrealistic assumption that we address by only applying the behavioural change twice, assuming that the existing SIF users will simply use the greater number of SIFs more frequently.

In order to calculate the economic benefits of reducing the number of HIV and HCV infections, values for the costs associated with these infections must be used. The life-time cost savings made from averted cases of HIV are at a large magnitude and range from CDN $70,000 to $25000 after considering the very successful multidrug combinations Highly Active Antiretroviral Therapy (HAART) [35-37]. Though the HAART treatments are highly effective, they are rather intensive and have low adherence rates within the PWID population [38]—Laufer [39] has argued that the PWID population is less likely to take full advantage of the medical system. We chose to follow the recent research by Pinkerton [16,17], who used CDN$ 210 555, based on the work of Albert et al. [25].

With respect to HCV, the most recent costing studies range from CAN$20,000 to CDN$30,000 [40] to more than CDN$69,188 [41], per completed patient course of treatment [42]. In the current analysis, we follow the National Centre in HIV Epidemiology and Clinical Research [43], using CDN$35,143 (2013 Dollars). We use this figure for a more conservative estimate regarding the complications arising from HCV, disregarding the costs for liver failure, hepatocellular carcinoma and liver transplant cases.

It is important to note that the calculated cost-savings of Insite are an under-estimate of the actual cost-savings. In our analyses, we do not consider any growth of the PWID population [15], new secondary HIV and HCV infections [44,45], or any reductions in other harms such as cellulitis, subcutaneous abscesses, endocarditis, and other soft-tissue infections [44]. Perhaps, more significant is the fact that we do not consider the value of a prevented death. Though the economic benefits are significant, previous research has found that few deaths are actually prevented from the Vancouver SIF, Insite [8]. This is also a contentious socio-political issue that we decided to avoid in the current analysis. Consequently, all cost-savings are more or less an underestimation when we consider the actual cost-savings.

In order to calculate cost-benefit ratios, the total operational costs of a SIF must be known. We use the cost for the Vancouver SIF as a proxy: CDN$3 million [16,17,46]. This is the cumulative cost for Insite that includes the SIF, addiction counselling and case management, the provision of primary healthcare, public health screening (immunisations and diagnostics), addiction and housing services, education, and peer counselling. Andresen and Boyd [8] list the annual operational cost of the SIF portion of Insite as CDN$1.5 million, CDN$2.183 million for a 24-hour operation in current dollars [47]. This is the figure that we use because we are only considering the establishment of a SIF in Ottawa. We acknowledge that this figure is likely to be an overestimatation of the total operational costs of a SIF in Ottawa because a SIF in Ottawa would likely be smaller in scale than in Vancouver. However, we are unaware of any corresponding data that could be used for such a cost estimate in Ottawa and, as such, the operational costs used here adds to the conservative nature of our estimates.

## Results and discussion

Results of the current study focus on the cost-benefits and cost-effectiveness of proposed SIFs in Ottawa, Ontario. These results are based solely on the prevention of new HIV and HCV cases, taking into account needle sharing rates and the PWID behavioural changes that would occur outside the SIFs. The results presented in Tables 3 and 4 show that the establishment of SIFs in Ottawa would result in a decrease in the number of new HIV and HCV cases. Specifically, the cumulative annual cost model (Table 3) indicates that 5 to 19 HIV cases may be averted while 48 to 191 HCV cases may be averted depending on the number of SIFs established. The prevention impact of the marginal annual cost model (Table 4) is not nearly as powerful with ranges of 2 to 5 and 21 to 48 cases of HIV and HCV being averted, respectively.

**Table 3:** The cumulative annual cost - effectiveness and benefit-cost of SIF in Ottawa using the first model

**Variables**	**Annual cost of operation**	**Sharing rate**	**# of HIV averted**	**# of HCV averted**	**Cost-effectiveness ratio HCV**	**Cost-effectiveness ratio HIV**	**Benefit-cost ratio HCV**	**Benefit-cost ratio HIV**	**Cost-benefit ratio Total**
Post SIF	$2,182,800	11%	5	48	$45,475	$436,560	0.77	0.48	1.26
		**(14%, 7%)**	**(6, 3)**	**(65, 31)**	**($33,581, $70,413)**	**($363,800, $727,600)**	**(1.1, 0.5)**	**(0.58, 0.3)**	**(1.63, 0.79)**
Two SIF	$4,365,600	8%	9	88	$49,609	$485,067	0.71	0.43	1.14
		**(11%, 5%)**	**(12, 6)**	**(120, 57)**	**($36,380, $76,589)**	**($383,800, $727,600)**	**(1, 0.46)**	**(0.58, 0.3)**	**(1.54, 0.75)**
Three SIF	$6,548,400	6%	11	112	$58,468	$595,309	0.6	0.35	0.95
		**(9%, 4%)**	**(15, 7)**	**(148, 70)**	**($44,246, $93,549)**	**($436,560, $936,486)**	**(0.8, 0.38)**	**(0.48, 0.22)**	**(1.28, 0.6)**
Four SIF	$8,731,200	5%	13	129	$67,683	$671,631	0.52	0.31	0.83
		**(7%, 3%)**	**(17, 8)**	**(175, 83)**	**($49,893, $105,195)**	**($513,600, $1,091,400)**	**(0.7, 0.33)**	**(0.41, 0.19)**	**(1.11, 0.5)**
Five SIF	$10,914,000	3%	15	150	$72,760	$727,600	0.48	0.29	0.77
		**(5%, 2%)**	**(20, 9)**	**(203, 96)**	**($53,764, $113,688)**	**($545,700, $1,212,667)**	**(0.65, 0.31)**	**(0.39, 0.17)**	**(1.04, 0.48)**
Six SIF	$13,096,800	2%	17	170	$77,040	$770,400	0.46	0.27	0.73
		**(3%, 1%)**	**(23, 10)**	**(232, 110)**	**($569,426, $119,062)**	**($569,426, $1,309,680)**	**(0.62, 0.3)**	**(0.37, 0.16)**	**(0.99, 0.46)**
Seven SIF	$15,279,600	1%	19	191	$79,998	$804,189	0.44	0.26	0.70
		**(1%, 1%)**	**(26, 12)**	**(259, 123)**	**($58,995, $124,224)**	**($587,677, $1,273,300)**	**(0.6, 0.28)**	**(0.36, 0.16)**	**(0.55, 0.45)**

Note: The numbers in parentheses represent the results of the sensitivity analysis: (19 per cent sharing rate, 9 percent sharing rate).

**Table 4:** The marginal annual cost - effectiveness and benefit-cost of sif in Ottawa using the first model

**Variables**	**Annual cost of operation**	**Sharing rate**	**# of HIV averted**	**# of HCV averted**	**Cost-effectiveness ratio HCV**	**Cost-effectiveness ratio HIV**	**Benefit-cost ratio HCV**	**Benefit-cost ratio HIV**	**Cost-benefit ratio Total**
**Post SIF**	**$2,182,800**	11%	5	48	$45,475	$436,560	0.77	0.48	1.26
		**(14%, 7%)**	**(6, 3)**	**(65, 31)**	**(33,581, $70,413)**	**($363,800, $727,600)**	**(1.1, 0.5)**	**(0.58, 0.3)**	**(1.63, 0.79)**
**Two SIF**	**$2,182,800**	8%	4	41	$53,239	$545,700	0.66	0.39	1.05
		**(11%, 5%)**	**(6, 3)**	**(55, 26)**	**($39,687, $83,954)**	**($363,800, $727,600)**	**(0.89, 0.42)**	**(0.58, 0.3)**	**(1.46, 0.71)**
**Three SIF**	**$2,182,800**	6%	2	24	$90,950	$1,091,400	0.39	0.19	0.58
		**(9%, 4%)**	**(3, 1)**	**(28, 14)**	**($77,957, $155,914)**	**($727,600, $2,182,800)**	**(0.45, 0.23)**	**(0.29, 0.1)**	**(0.74, 0.32)**
**Four SIF**	**$2,182,800**	5%	2	17	$128,400	$1,091,400	0.27	0.19	0.47
		**(7%, 3%)**	**(3, 1)**	**(28, 13)**	**($77,957, $167,908)**	**($727,600, $2,182,800)**	**(0.45, 0.21)**	**(0.29, 0.1)**	**(0.74, 0.31)**
**Five SIF**	**$2,182,800**	3%	2	21	$103,943	$1,091,400	0.34	0.19	0.53
		**(5%, 2%)**	**(3, 1)**	**(28, 13)**	**($77,957, $167,908)**	**($727,600, $2,182,800)**	**(0.45, 0.21)**	**(0.29, 0.1)**	**(0.74, 0.31)**
**Six SIF**	**$2,182,800**	2%	2	21	$103,943	$1,091,400	0.34	0.19	0.53
		**(3%, 1%)**	**(3, 1)**	**(28, 13)**	**($77,957, $167,908)**	**($727,600, $2,182,800)**	**(0.45, 0.21)**	**(0.29, 0.1)**	**(0.74, 0.31)**
**Seven SIF**	**$2,182,800**	1%	2	21	$103,943	$1,091,400	0.34	0.19	0.53
		**(1%, 1%)**	**(3, 1)**	**(28, 13)**	**($77,957, $167,908)**	**($727,600, $2,182,800)**	**(0.45, 0.21)**	**(0.29, 0.1)**	**(0.74, 0.31)**

Note: The numbers in parentheses represent the results of the sensitivity analysis: (30 per cent sharing rate, 10 percent sharing rate).

With respect to the fiscal implications of these results, the decrease in HIV and HCV cases are not enough to independently cover the cost of SIF operations. In fact, when considering the operation of the first two SIFs in Tables 3 and 4, where behavioural change impacts the rate of needle sharing, both the cumulative and marginal cost-benefit ratios are below unity. Specifically, the cumulative cost-effectiveness for HIV cases (Table 3) ranges from CDN$436,560 to CDN $804,189 where the costs associated with a single HIV case is CDN$210, 555. The cumulative cost-effectiveness for HCV (Table 3) ranges from CDN $45,475 to CDN $79,998 where the cost of a single HCV case is CDN$35,143. Both of these ratios are far above the estimated cost per HIV and HCV case resulting in cost-benefit ratios below 1.0. However, when the cost-benefit ratios considering both HIV and HCV are considered simultaneously, there is a financial justification for at least two SIFs, if not three SIFs with the last cost-benefit-ratio being 0.95—close enough to 1.0 in this conservative modeling methodology. This highlights the importance of considering the additive effects of HIV and HCV from the establishment of a SIF. In fact, as can be seen in Table 3, the driver of the cost savings in these models is HCV, ignored by many of the recent cost evaluations of the Vancouver SIF. However, as indicated before, owing to the lack of a definitive demonstration of a relationship in the scientific literature, the result that HCV is a driving factor in the cost saving must be interpreted with caution.

Others have also estimated decreases in HCV from SIFs [21], but this was derived from a mathematical model.

The independent marginal cost-effectiveness for both HIV and HCV (Table 4) are also far above the estimated cost per HIV and HCV case. The marginal cost-effectiveness ranges from CDN$436,560 to CDN$1,091,400 for HIV and from CDN$45,475 to CDN$103,943 for HCV. Again, with costs associated with an HIV case set at CDN$210, 555 and an HCV case set at CDN$35,143, cost-benefit ratios are below 1.0 and thus, the models do not support the establishment of SIFs when HIV and HCV are considered independently. But from a total cost-benefit perspective, two SIFs can be justified when considering their marginal impacts on HIV and HCV.

A sensitivity analyses conducted at different baseline sharing rates (9 and 19 per cent), however, demonstrates that changes to the needle sharing rates influence the results in an important way (see Tables 3 to 4). Specifically, the cumulative (Table 3) and marginal (Table 4) annual cost models with 9% sharing rates do not support the establishment of any SIFs as cost-benefit ratios are all below unity. The cost-effectiveness ratios for HIV and HCV cases, however, support the establishment of as many as five (or even six) SIFs when the sharing rate is set at 19% (see Tables 3 and 4). However, if one were to only consider HIV or HCV independently, the establishment of a SIF would only be considered as “cost saving” in the case of HCV, with a maximum of two SIFs. The marginal annual cost model with a 19% sharing rate supports the establishment of a single SIF with a cost-benefit ratio of 1.1 for only HCV. However, it accounts for two SIFs when considering the additive impact of HIV and HCV. Given that the baseline sharing rate of 14% used here is likely to be an underestimation, it can be argued that the establishment of SIFs should be given serious consideration^a^.

Finally, Tables 5 present results of the cost-effectiveness and cost-benefit of proposed SIFs using the Kaplan and O’Keefe [5] model that focuses on prevented HIV cases. As is evident from this table, the number of HIV cases prevented is not enough to cover the cost of operating a SIF. Moreover, the cumulative and marginal cost-benefit ratios are below 1.0 in all SIF scenarios. The same may be said for the marginal and cumulative cost-effectiveness ratios.

**Table 5:** The Cumulative and Marginal Cost - Effectiveness and Cost – Benefit of SIF in Ottawa Using the Second Model

**Variables**	**Annual cost of operation**	**Sharing rate**	**#of HIV averted**	**Cost-effectiveness ratio HIV**	**Benefit-cost ratio HIV**
**Post SIF**	$2,182,800	11%	7	$311,829	0.68
	**($2,182,800)**		**(7)**	**($311,829)**	**(0.68)**
**Two SIF**	$4,365,600	8%	13	$335,815	0.63
	**($2,182,800)**		**(6)**	**($363,800)**	**(0.6)**
**Three SIF**	$6,548,400	6%	16	$409,275	0.51
	**($2,182,800)**		**(3)**	**($727,600)**	**(0.3)**
**Four SIF**	$8,731,200	5%	18	$485,067	0.43
	**($2,182,800)**		**(2)**	**($1,091,400)**	**(0.19)**
**Five SIF**	$10,914,000	3%	21	$519,714	0.4
	**($2,182,800)**		**(3)**	**($727,600)**	**(0.3)**
**Six SIF**	$13,096,800	2%	24	$545,700	0.38
	**($2,182,800)**		**(3)**	**($727,600)**	**(0.3)**
**Seven SIF**	$15,279,600	1%	27	$565,911	0.37
	**($2,182,800)**		**(3)**	**($727,600)**	**(0.3)**

Note: The numbers in parentheses represent the marginal results.

## Conclusions

Several studies have demonstrated the fiscal advantages of operating Insite – North America’s only legal SIF [8,14-17]. Research into the economic viability of expanding SIFs to other locations, however, is still in its infancy. The current study was aimed at contributing to that growing body of literature, by conducting cost-benefit and cost-effectiveness analyses for the opening of SIFs in Ottawa, Ontario. Specifically, the costs of operating various numbers of SIFs in Ottawa was compared to the savings incurred after accounting for the prevention of new HIV and HCV infections.

Results of this study revealed that according to several analyses, there is an economic incentive to operating SIFs in Ottawa only if both HIV and HCV are considered. This is of importance because the specific PWID characteristics vary in different areas. As a result of this, different base rates of HIV and HCV infection are likely to determine whether SIFs are cost-effective or not. The independent analyses of HIV and HCV, for their cumulative and marginal annual cost analyses, using the Jacob’s et al. [31] model both revealed a maximum cost-benefit ratio of 0.48 for HIV cases and 0.77 for HCV cases. Although a slight improvement over those results, the Kaplan and O’Keefe [5] cumulative and marginal annual cost models also fell short of positive results with a cost-benefit ratio of 0.68 for HIV cases. Only when both the effects of reduced HIV and HCV infections were considered, did the establishment of SIFs achieve cost benefits.

The sensitivity analyses conducted with the first model did, however, reveal the potential for SIFs in Ottawa to be a fiscally responsible harm reduction strategy for the prevention of HCV cases – when considered independently. With a baseline sharing rate of 19%, the cumulative annual cost model supported the establishment of two SIFs and the marginal annual cost model supported the establishment of a single SIF. The cumulative annual cost model that considered both HIV and HCV, however, could justify as many as six SIFs while the marginal annual cost model supported the establishment of two SIFs. Though these results rely on a needle sharing rate that is higher than the conservative baseline rate used in the other analyses, serious consideration should be given to the establishment of SIFs in Ottawa; especially since other studies have demonstrated that the 14% baseline rate is an underestimated rate of needle sharing in the city rather than overestimated one.

These results also demonstrate the need to routinely collect accurate, up-to-date, and geographically specific data so that studies such as this may help to inform public policy with greater accuracy and confidence. Moreover, these results also show the importance of considering more than one potential benefit in cost-benefit analyses for public health. Though the cost savings from one averted HCV case would be considerably lower than that of an averted HIV case (16.69%), the volume of averted cases of HCV is able to significantly impact cost savings. Consequently, if we are to properly assess the impact of harm reduction strategies on our health care system, we must be as inclusive as possible regarding potential benefits in order to identify all possible savings. In moving forward, research should also consider how to facilitate the implementation of new SIFs.

We must emphasize that the largest obstacle to implementing a SIF in Ottawa is strong opposition from the local municipal government and police force as well as the federal government. These factors are likely to preclude the opening of a SIF in Ottawa irrespective of scientific evidence supporting the implementation of this intervention. The local health officials, not the federal or provincial government, should make decisions regarding opening SIFs, based on the positive impact of SIFs in reducing injections in public, while lowering the overdose fatalities and infectious diseases [8-11]. SIFs have not increased crime, drug dealing, public injection, public syringe disposal, neither have they contributed to disturbing public order [48-50]. Accordingly, “concerns that arise out of prejudice and ignorance for which there are no sound arguments should be set aside” [51], p. 1304. This will ultimately help in conceptualizing the injection drug use as a public health issue, rather than a moral one.

## Endnote

^a^The baseline sharing rate of 14% is deemed likely to be an underestimate given that some studies have found much higher rates of needle sharing in Ottawa. In Leonard et al. [52], for example, 37% of female PWID and 31% of male PWID’s reported injecting with previously used needles within the previous six months. In Leonard et al. [53], 27% of female and 19% of male PWID reported that they had indulged in using shared needles in the previous six months. For the current study, however, the most conservative (under-) estimate of needle sharing is used as a baseline value (14%).

## Abbreviations

HCV: Hepatitis C; PWID: People who Inject drugs; SIF: Supervised injection facility; HIV: Human immunodeficiency virus.

## Competing interests

There are no competing interests to report.

## Authors’ contributions

EJ collected the data and conducted the analysis, AAR wrote the result, discussion and conclusion and references, MAA wrote the related studies section, methods and reviewed the analysis, and AJ wrote the introduction and references. All authors read and approved the final version of the paper.
